# Distinct retinoic acid receptor (RAR) isotypes control differentiation of embryonal carcinoma cells to dopaminergic or striatopallidal medium spiny neurons

**DOI:** 10.1038/s41598-017-13826-x

**Published:** 2017-10-20

**Authors:** Anna Podleśny-Drabiniok, Joanna Sobska, Angel R. de Lera, Krystyna Gołembiowska, Katarzyna Kamińska, Pascal Dollé, Małgorzata Cebrat, Wojciech Krężel

**Affiliations:** 1 Institut de Génétique et de Biologie Moléculaire et Cellulaire, Illkirch, France; 2 Institut de la Santé et de la Recherche Médicale, U964 Illkirch, France; 3 Centre National de la Recherche Scientifique, UMR 7104 Illkirch, France; 4 Université de Strasbourg, Illkirch, France; 5 Laboratory of Molecular and Cellular Immunology, Department of Tumor Immunology, L. Hirszfeld Institute of Immunology and Experimental Therapy, Polish Academy of Sciences, Weigla 12, 53-114 Wroclaw, Poland; 6 Advanced Materials Engineering and Modelling Group, Faculty of Chemistry, Wroclaw University of Science and Technology, Wyb. Wyspianskiego 27, 50-370 Wroclaw, Poland; 7 Departamento de Química Orgánica, Facultade de Química, CINBIO and IIS Galicia Sur, Universidade de Vigo, Vigo, Spain; 8 Department of Pharmacology, Institute of Pharmacology, Polish Academy of Sciences, Kraków, Poland

## Abstract

Embryonal carcinoma (EC) cells are pluripotent stem cells extensively used for studies of cell differentiation. Although retinoic acid (RA) is a powerful inducer of neurogenesis in EC cells, it is not clear what specific neuronal subtypes are generated and whether different RAR isotypes may contribute to such neuronal diversification. Here we show that RA treatment during EC embryoid body formation is a highly robust protocol for generation of striatal-like GABAergic neurons which display molecular characteristics of striatopallidal medium spiny neurons (MSNs), including expression of functional dopamine D2 receptor. By using RARα, β and γ selective agonists we show that RARγ is the functionally dominant RAR in mediating RA control of early molecular determinants of MSNs leading to formation of striatopallidal-like neurons. In contrast, activation of RARα is less efficient in generation of this class of neurons, but is essential for differentiation of functional dopaminergic neurons, which may correspond to a subpopulation of inhibitory dopaminergic neurons expressing glutamic acid decarboxylase *in vivo*.

## Introduction

Retinoic acid (RA), the bioactive form of vitamin A, is essential for embryonic development by virtue of controlling proliferation, differentiation and homeostasis of diverse cell types, thus contributing to organogenesis and formation of different types of tissues including nervous tissue. Research carried out over more than 30 years provided examples of critical roles of RA in generation of diverse neural cell types and neuronal subtypes in different regions of the nervous system (reviewed in ref.^1–4^). Notably, at early stages of development, RA instructs neuroectoderm differentiation towards neuronal progenitors and thus controls formation of generic neurons in future hindbrain and spinal cord^5–7^. At later phases of development, RA also contributes to the diversification of neuronal subtypes, as best illustrated for generation of motor neuron subtypes^8–11^ and formation of serotonergic^12,13^ and noradrenergic neurons^14^. The potential involvement of RA in development of midbrain dopaminergic neurons (mDA) was also suggested by RA-dependent prevention of stark deficits of mDA cell development in Pitx3−/− mice^15^. In the forebrain, although RA is not essential for generation of neural progenitor cells at early stages of development, it has been reported to be an important determinant of neuronal differentiation. Some of the most striking effects of RA deficiency were observed in the lateral ganglionic eminence (LGE), a transient structure from which the striatum will develop. In the absence of retinaldehyde dehydrogenase 3 (Raldh3), the major RA-synthesizing enzyme in this region, differentiation of projection medium spiny neurons (MSNs) and interneurons was almost completely abrogated and such effect was best visible in primary neuronal cultures from Raldh3−/− LGE^16^. Although RARα and RARβ are the only two RARs which could mediate such RA activity (RARγ is not expressed in the LGE)^17–19^, genetic ablation of RARβ in mice affected development of only striatonigral projection neurons, a subpopulation of MSNs expressing dopamine receptor Drd1, and did not affect generation of striatopallidal MSNs expressing Drd2^19,20^. These two populations of inhibitory, GABAergic neurons define two main output pathways of the striatum, and their unbalanced signaling is at the origin of physiopathology of several disorders of basal ganglia^21^. These data indicate that although RA controls differentiation of an overall population of GABAergic neurons in the striatum, distinct RARs (or their sequential activities) may differentially contribute to formation of specific neuronal subtypes.

Knowledge of the key endogenous determinants of neural progenitor induction and neuronal subtype specification is critical for establishing protocols for generating specific neuronal types *in vitro*, with an ultimate goal of their use for cell replacement and regenerative strategies in treatment of neurodegenerative diseases. Some of these key neurodevelopmental signals, including secretory molecules (e.g. all-*trans*-RA [ATRA], FGF or Wnt), together with intrinsic signals (e.g. specific transcription factors), were used to induce undifferentiated mouse and human embryonic stem (ES) cells to neurons with different subtype characteristics. Treatments with ATRA and Sonic hedgehog^22–25^ were used to generate different subtypes of motor neurons, whereas ATRA application alone or in combination with diverse additional factors were used to generate dopaminergic^26,27^, cholinergic, glutamatergic^28^ or GABAergic neurons^16,29–31^. Some of the generated GABAergic neurons displayed striatal-like phenotypes^16^ with exception of striatal projection neurons^16,32^.

Pluripotent embryonal carcinoma (EC) stem cells closely resemble embryonic stem cells. Since its isolation in the early 1980’s^33^, the mouse P19 EC cell line has been an attractive model used to study the mechanisms controlling neurogenesis. With the recent advances in genomic technologies, many genes and signaling pathways involved in programming and reprogramming towards neuronal cells were identified using distinct differentiation protocols^34–36^. However, despite initial efforts to characterize cell types generated from EC cells in the 80’s and early 90’s, the specific subtypes of GABAergic neurons induced by retinoids from EC cells remain largely unknown. By using lineage specific markers and/or biochemical analyses, some of the original studies reported the possibility of obtaining mostly neurons, out of which few were cholinergic^37^. GABAergic inhibitory neurons expressing glutamic acid decarboxylase (GAD65/67) were found the most frequently, but lower numbers of somatostatin- or NPY-expressing neurons were also identified^38^. In the same study, few cells expressing catecholaminergic markers including tyrosine hydroxylase (TH) were also identified, which could correspond to noradrenergic neurons, as dopamine was never detected in those cells. Differences in culture conditions including duration of embryoid body formation prior and after ATRA treatments, ATRA concentrations, types of plate coatings or duration of cultures make comparisons between different reports difficult, although collectively these studies highlighted robustness of EC cells to generate neurons and minor populations of other cell types including microglia cells^39^, oligodendrocytes^40^ or GFAP^+^ astrocytes^37^. Neurons obtained from EC cells were shown to form functional synaptic connections^38,41^, which stimulated trials of their use for cell replacement therapies in animal models of neurodegenerative diseases^42,43^.

Despite the demonstration of a critical role of ATRA in differentiation of EC cells to neurons, the contribution of individual retinoid acid receptors (RARα, RARβ and RARγ) in this process is not known. It is possible that distinct RAR isotypes may differentially contribute to generating cellular and/or neuronal diversity during EC differentiation. This possibility is supported by a recent study indicating that activation of distinct receptors induces distinct differentiation programs^34^. In the present work, by using synthetic retinoids that act as RAR-selective agonists, we have dissected the contributions of each type of RAR to differentiation of neuronal populations in differentiating P19 cells grown in suspension (embryoid bodies). We demonstrate that ATRA promotes robust differentiation of EC cells into GABAergic, DARPP32-positive striatal-like medium spiny neurons (MSNs) expressing key markers of striatopallidal projection neurons including dopamine receptor D2 (Drd2), adenosine receptor 2a (Adora2a) and proenkephaline (pEnk). We also show that RARγ is functionally predominant in such control, whereas RARα promotes differentiation of a sub-population of dopamine-producing neurons expressing tyrosine hydroxylase (TH) and dopamine transporter (DAT). Interestingly, these neurons are also GABAergic and may correspond to the small (10%) population of dopaminergic neurons which express also GAD65/67 in substantia nigra^44^. Neither the function nor the developmental origin of these neurons is known.

## Results

### Retinoic acid differentiates EC cells into GABAergic, striatopallidal-like medium spiny neurons

**Figure 1:**
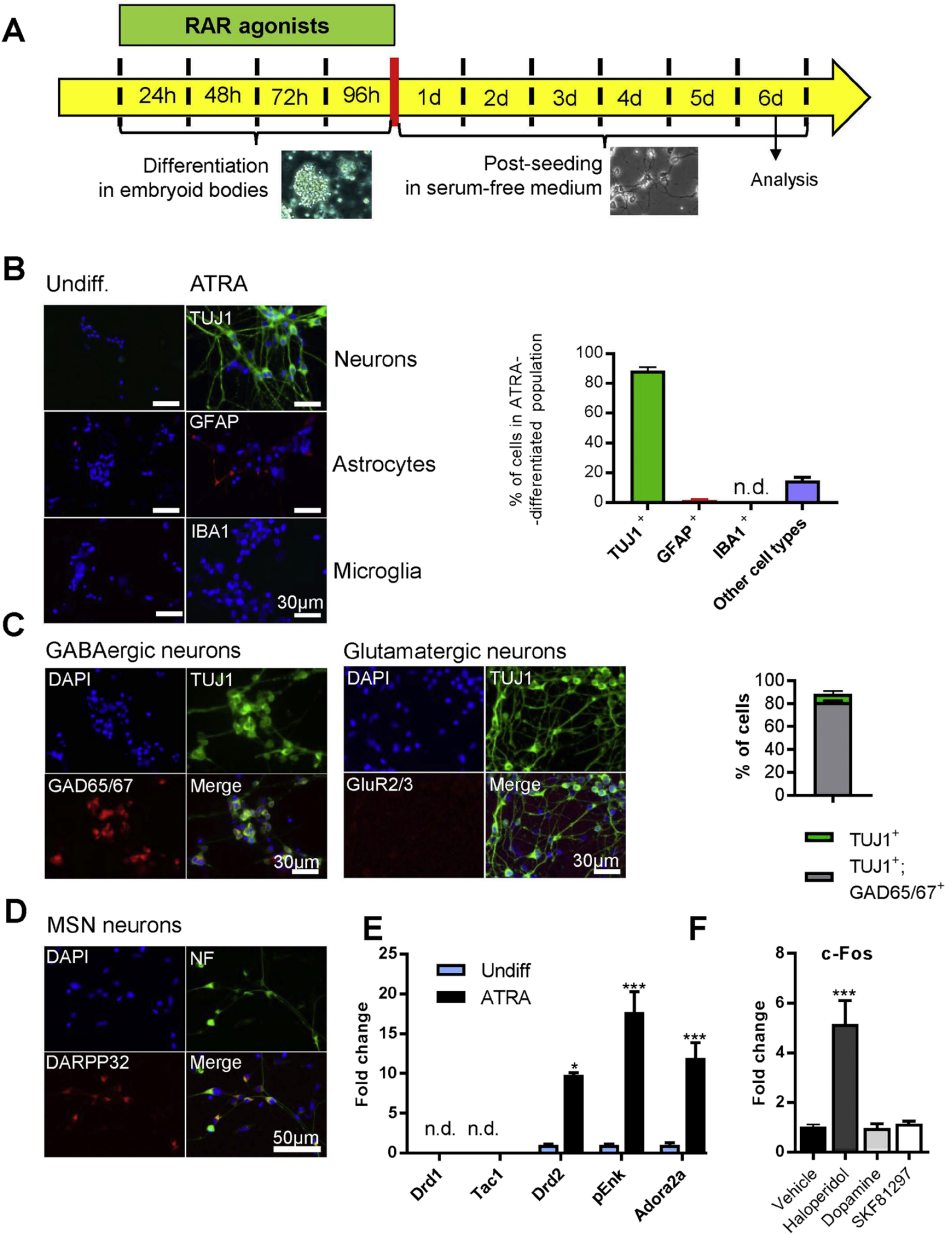
Cellular populations after ATRA-mediated differentiation of EC cells. (**A**) Experimental design of embryoid body differentiation, retinoid treatments, and sample collection. (**B**) Neurons, astrocytes and microglial cells were assessed in ATRA-differentiated EC cells by immunofluorescent detection of TUJ1, GFAP and IBA1, respectively (see left panels for an example, and histograms for quantification). (**C**) Immunofluorescence analyses of excitatory glutamatergic (GluR2/3^+^) and inhibitory (GAD65/67^+^) neurons. (**D**) Immunofluorescent detection of DARPP32^+^ medium-spiny neurons (MSNs) within neuronal (Neurofilament, NF^+^) cells. (**E**) mRNA expression levels of striatonigral and striatopallidal MSN markers quantified by qPCR. Expression is plotted as fold change as compare to expression level in undifferentiated cells. *p < 0.05; **p < 0.01; ***p < 0.001 as compare to undifferentiated cells. (**F**) Induction of c-Fos expression measured by RT-qPCR, 90 min after haloperidol (10 µM), dopamine (1 µM) or SKF81297 (1 µM) treatment. Expression is plotted as a fold change with respect to control cells treated with vehicle. ***p < 0.001 as compared to vehicle treatment. Throughout the figure, graph bars represent mean ± s.e.m. for 3 independent experiments for immunocytochemistry and 5 independent experiments for qPCR. n.d., not detected.

To address the role of ATRA in generation of specific neuronal types and subtypes, we used a differentiation protocol applied recently to dissect genetic programs underlying ATRA-induced neurogenesis in EC cells^35^. Differentiation of EC aggregates was induced by ATRA, or selective agonists of RARα (BMS753), RARβ (BMS641), or RARγ (CD666). The cell types generated were determined by analyses of gene (RT-qPCR) and protein expression (immunocytochemistry and flow cytometry) of selected markers, 6 days after the end of retinoid treatment and post-seeding in serum-free medium (Fig. 1A).

Immunocytochemistry analyses revealed that following ATRA treatment, 88.5 ± 2.5% cells expressed a pan-neuronal marker, beta III-tubulin (TUJ1 antibody), indicating that neurons are the major cell type obtained after ATRA-induced differentiation (Fig. 1B). Indeed, only few astrocytes (1,5 ± 0.4% of all cells) were identified by expression of glial fibrillary acidic protein (GFAP) and no microglia (IBA1^+^) was detected. This observation was further supported by quantification of corresponding transcript levels (Supplemental Fig. 1A). We found that a vast majority of neurons (81.0 ± 1.3% of all cells and 90% of TUJ1^+^ cells) were inhibitory and expressed glutamate decarboxylase 65/67 (GAD65/67), whereas analyses of glutamate receptor 2/3 (GluR2/3) expression showed no evidence of excitatory glutamatergic neurons (Fig. 1C).

GABAergic inhibitory neurons are highly diverse and their molecular, electrophysiological and morphological characteristics change across different structures^45–47^. In the striatum, medium spiny GABAergic neurons are the major neuronal type and their generation was shown to critically depend on RA signaling *in vivo* ^16^. We found that more than 90% neurons obtained after RA treatment of EC cells (Fig. 1D) express protein phosphatase 1 regulatory subunit 1B (PPP1R1B, also known as DARPP32), which is strongly enriched in MSNs, suggesting a striatal-like identity of these neurons. To further test this possibility, we analyzed expression of dopamine receptors Drd1 and Drd2, which determine, respectively, striatonigral and striatopallidal projection MSNs constituting the two main output pathways of the striatum. Using RT-qPCR we found only Drd2, but no Drd1 expression, indicating that EC cells differentiated to Drd2^+^ striatopallidal MSNs. Accordingly, obtained neurons expressed adenosine A2a receptor (Adora2a) and proenkephalin (pEnk), distinct markers of this MSN subtype, but were devoid of Tachynin1 (Tac1) and Drd1 found in striatonigral MSNs (Fig. 1E). Drd2 expressed in such MSN-like cells was functional, as illustrated by a significant increase of c-Fos expression following inhibition of Drd2 signaling by haloperidol (10 µM) treatment. No induction of c-Fos expression was observed after dopamine (1 µM) or SKF81297 (Drd1 specific agonist; 1 µM) treatment, indicating absence of functional D1-type dopamine receptors (Fig. 1F).

### Retinoic acid receptors promote differentiation of distinct subtypes of GABAergic neurons

**Figure 2:**
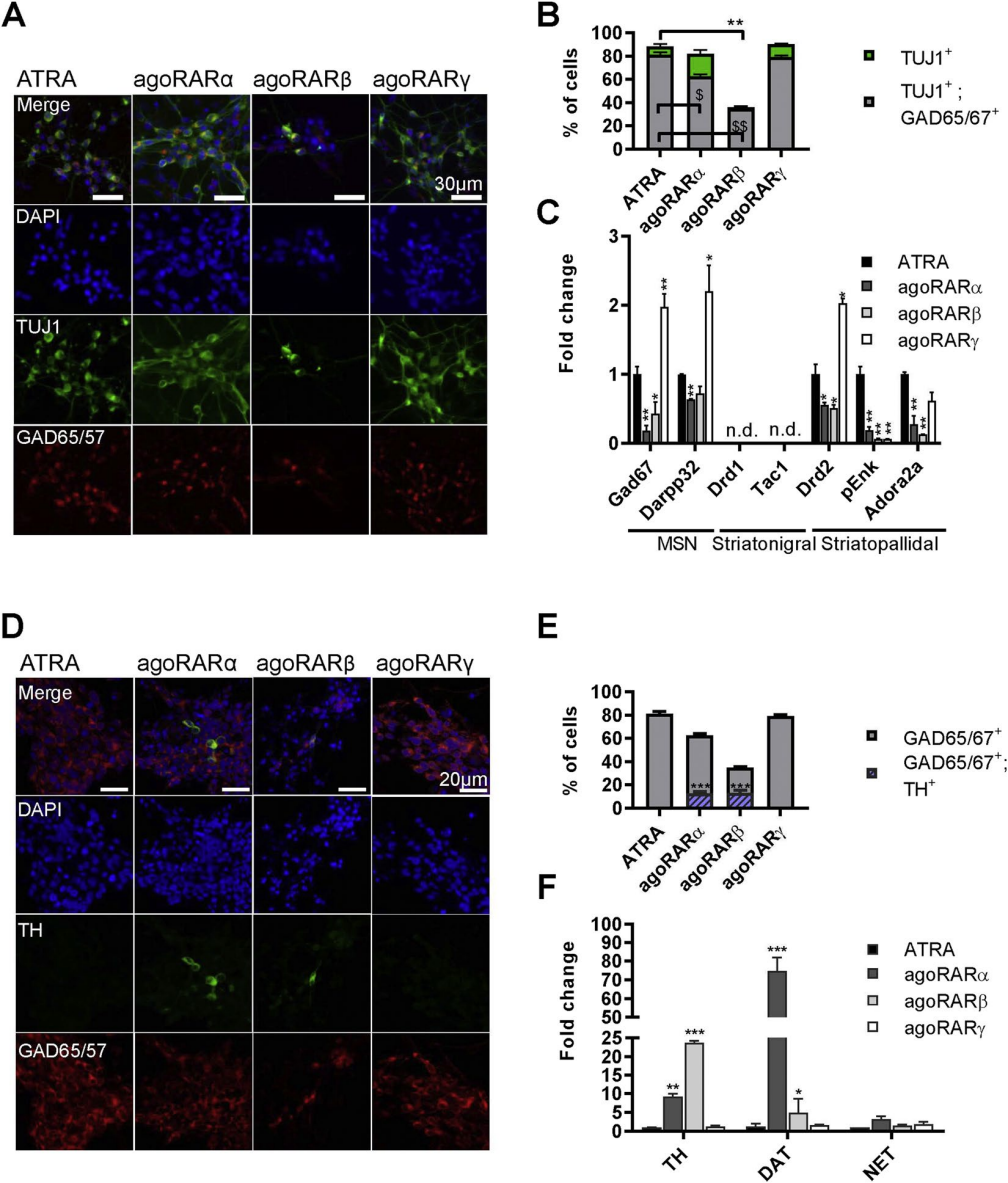
Differentiation of EC embryoid bodies by distinct RAR agonists. (**A**) Examples of immunofluorescent detection of neuronal (TUJ1^+^) and GABAergic (GAD65/67^+^) markers in cell populations obtained after treatments with different RAR agonists. (**B**) Quantification of GABAergic (TUJ1^+^; GAD65/67^+^) and non-GABAergic neurons (TUJ1^+^) (n = 4 experiments per treatment). **p < 0.01 as compare to TUJ^+^ cells obtained in ATRA treatment; ^$^p < 0.05, ^$$^p < 0.01 as compare to number of GAD65/67^+^ neurons obtained after ATRA differentiation (**C**) mRNA expression levels of striatonigral and striatopallidal MSN markers quantified by qPCR for each treatment group (n = 5 experiments). The expression of each marker is plotted as fold change with respect to ATRA treatment. *p < 0.05; **p < 0.01 as compared to expression level in ATRA group. (**D**) Example of immunofluorescent detection of GABAergic (GAD65/67) and dopaminergic (TH) markers in cell populations obtained after treatment with selective RAR agonists. (**E**) Quantification of GAD65/67^+^ and GAD65/67^+^; TH^+^ neurons (n = 4 experiments) ***p <0.001 as compared to ATRA group. (**F**) mRNA expression of markers specific for dopaminergic and noradrenergic neurons (n = 5 experiments). The expression of each marker is plotted as fold change with respect to ATRA treatment. ***p < 0.001,**p < 0.01, *p < 0.05 as compared to ATRA group. n.d., not detected.

**Figure 3:**
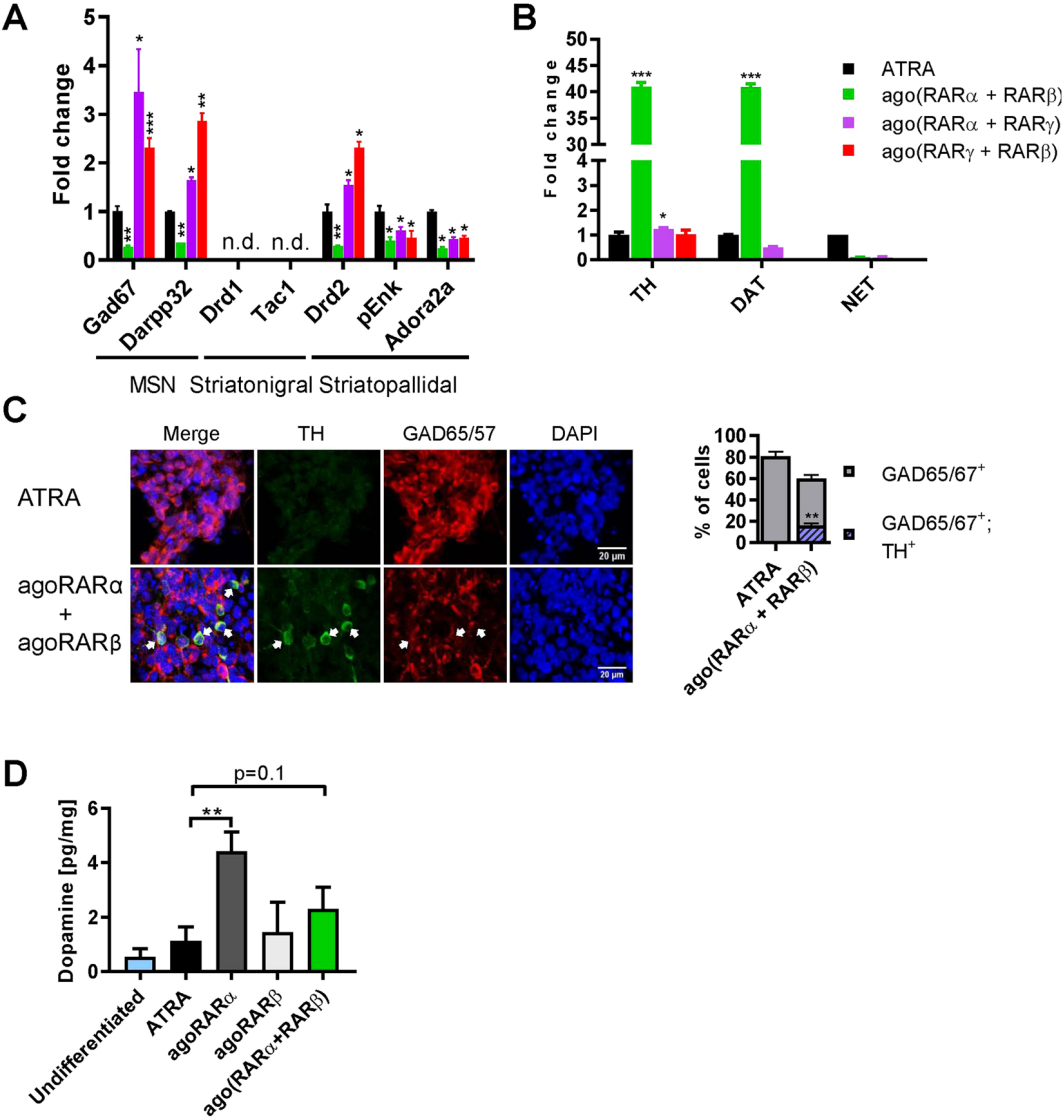
Differentiation of EC embryoid bodies by compound RAR agonist treatments. (**A**) qPCR quantification of relative mRNA levels of striatonigral and striatopallidal MSNs, and (**B**) dopaminergic and noradrenergic markers in neuronal population obtained after treatment with combination of two selective agonists. The expression of each marker was shown as fold change with respect to ATRA treatment, *p < 0.05; **p < 0.01; ***p < 0.001 as compared to ATRA group. (**C**) Left: immunofluorescent detection of dopaminergic (TH^+^) and GABAergic (GAD65/67^+^) neurons obtained after ATRA or compound RARα and RARβ treatments. Arrows indicate neurons expressing GAD65/67 and TH. Right, quantification of GAD65/67^+^, TH^+^, and double positive cells (GAD65/67^+^ TH^+^). **p < 0.01 with respect to ATRA group. (**D**) Dopamine detection by high-performance liquid chromatography in mature neuronal population (n = 3–4 experiments per treatment). **p < 0.01 with respect to  ATRA group.

To assess the contribution of specific RARs to ATRA-mediated neurogenesis, we induced differentiation of EC aggregates using selective RARα, β, or γ agonists, and compared the resulting neuronal populations with those obtained after ATRA treatment. We found that RARα and RARγ agonists were as efficient as ATRA to induce neuronal cells expressing TUJ1 (Fig. 2A,B), and that none of the specific agonists stimulated expression of glial cells markers (GFAP or Iba1) (Supplemental Fig. 1B). Following induction of RARγ by CD666, 76.9 ± 0.2% of all cells were GABAergic, which was comparable with the efficiency of ATRA-mediated induction (81.0 ± 1.3%) (Fig. 2A,B). CD666 exceeded ATRA in induction of striatopallidal MSN-specific transcripts including DARPP32 and Drd2, but not pEnk and Adora2a (Fig. 2C). The RARα agonist, BMS753, also induced differentiation of GABAergic neurons, but less efficiently than CD666, leading to generation of only 63.1 ± 1.0% of GABAergic neurons (Fig. 2A,B). Also, BMS753-induced neurons expressed significantly lower levels of MSN markers, in comparison with ATRA or CD666 induction (Fig. 2C). The RARβ agonist, BMS641, was the least efficient to induce neurogenesis, leading to 32.0 ± 0.2% of TUJ1^+^ cells and only 28.5 ± 0.4% of GABAergic neurons (Fig. 2B), and low expression of MSN markers (Fig. 2C). The efficiency and specificity of different retinoids to induce neuronal differentiation was also confirmed by fluorescence-activated cell sorting (Supplemental Fig. 2). Remarkably, RT-qPCR analyses revealed that none of the selective agonists induced Drd1 or Tac1, the markers of striatonigral MSNs, indicating that retinoids induce preferentially GABAergic neurons of striatopallidal-like subtype expressing DARPP32, Drd2, pEnk and Adora2a. Such MSN signature was weaker for pEnk and Adora2a expression after induction by RARα and RARβ agonists, despite the high percentage of generated TUJ1^+^ neurons. We therefore tested expression of markers of dopaminergic neurons, which are also known to express Drd2 or DARPP32. Tyrosine hydroxylase (TH), a rate limiting enzyme in the synthesis of catecholamines, was found to be expressed in 13% of all cells differentiated after RARα or RARβ agonist treatment (Fig. 2D,E). Expression of dopamine transporter (DAT, a marker of dopaminergic neurons), and lack of expression of noradrenaline transporter (NET, a marker of noradrenergic neurons), indicated a dopaminergic phenotype of TH^+^ cells (Fig. 2F), which was further supported by dopamine production as detected by HPLC analyses of cells generated after RARα agonist treatment (Fig. 3D). Interestingly all of the TH^+^ cells were also GABAergic (Fig. 3C), suggesting that these cells may correspond to a sub-population of inhibitory dopaminergic neurons found in dopaminergic nuclei^44^.

### RARγ is the functionally predominant receptor for determination of neuronal cell types in differentiating EC cells

To understand the differential involvement of RAR isotypes in generating TH^+^ dopaminergic neurons and Drd2^+^ MSNs, we re-evaluated expression of individual receptors in undifferentiated cells and at 24 h after treatment with ATRA or selective RAR agonists. Whereas RARγ was the major RAR expressed in undifferentiated EC cells, exceeding by about 10-fold levels of RARα and RARβ (Supplemental Fig. 3A), addition of ATRA led to a >100-fold increase of RARβ expression (Supplemental Fig. 3B), which thereby became the major RAR expressed in differentiating EC cells. Each of the selective RAR agonists also induced RARβ expression, indicating that each of the retinoid receptor isotypes is functional in undifferentiated cells despite low expression levels (Supplemental Fig. 3C). Concomitant activation of RARα and RARβ (in absence of RARγ agonist) led to only a weak, non-significant increase in the number of TH^+^ dopaminergic neurons (compare Fig. 3B,C and Fig. 2E,F) and did not significantly enhance induction of striatopallidal-like Drd2^+^ MSNs as compared to individual agonists (compare Figs 2C and 3A). This suggests that RARα and RARβ activate most probably the same differentiation program. In turn, concomitant activation of RARγ and RARα or RARγ and RARβ by corresponding agonists, always led to Drd2^+^ MSN neurons with very weak or no expression of dopaminergic markers, indicating an isotype-specific and dominant contribution of RARγ in inducing a Drd2^+^ MSN cell type, and specificity of RARα in generating TH^+^ dopaminergic neurons (Fig. 3A,B). The contribution of RARβ in production of dopaminergic neurons was not critical as dopamine production, when determined by HPLC, was significant only in EC cells differentiated with the RARα agonist, whereas cells generated by combined treatment with BMS753 and BMS641 displayed only a strong tendency to produce dopamine (Fig. 3D).

### Early determinants of dopaminergic and striatal Drd2^+^ neurons are differentially induced by RAR agonists

**Figure 4:**
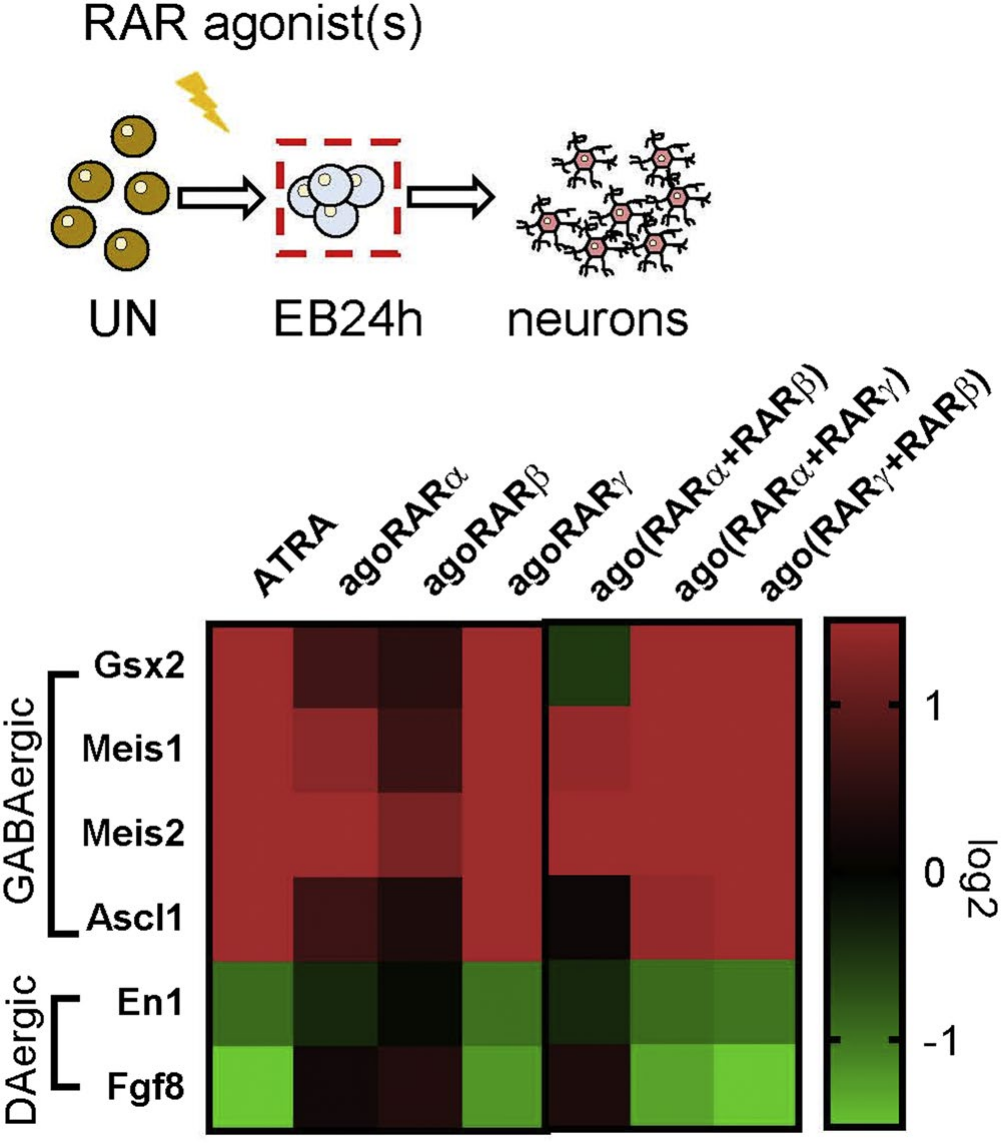
Neuron-specific programs at early stages of neuronal differentiation. (**A**) qPCR data of 24h-treated embryoid bodies (EB) are plotted as heat maps on a log_2_ scale (red, upregulated; green, downregulated, in comparison with expression values observed in undifferentiated EC cells). Each column corresponds to a specific retinoid treatment condition (n = 3–5 experiments/treatment).

To assess if induction of Drd2^+^ MSNs and DA neurons by ATRA or specific agonists is associated with early induction of developmental determinants of those cell types, we analyzed a selection of such molecular determinants at 24 h after the beginning of retinoid treatment (Fig. 4). We found that although most ligands induced Meis1 and Meis2, considered as important determinants of neurogenesis^34,48,49^, markers of GABAergic neurons and strongly enriched in developing striatum^19,50^, only ATRA and RARγ were highly efficient in induction of early determinants of GABAergic MSN development. Indeed, both ATRA and CD666 increased expression of Gsx2 and Ascl1, two main determinants of the lateral ganglionic eminence^51–53^, by 60- and 35-fold as compared to expression in undifferentiated EC cells (Fig. 4 and Supplemental Table 1). On the other hand, when compared to undifferentiated cells, ATRA and RARγ strongly suppressed expression of En1 (10-fold decrease) and Fgf8 (30-fold decrease), which during development are not expressed in the lateral ganglionic eminence but are important determinants of developing midbrain DA neurons^54,55^. Unlike the RARγ agonist, RARα and RARβ agonists led to only weak induction of Gsx2 (5- and 3-fold, respectively) and Ascl1 (5- and 2-fold, respectively), as compared to undifferentiated cells, whereas combined RARα and RARβ agonist treatment suppressed Gsx2 (3-fold decrease) and did not induce Ascl1 expression. Instead, both agonists (BMS753 and BMS641), individually or after combined application, induced Fgf8 expression and maintained high levels of En1 expression present already in undifferentiated cells (for exact values see Supplemental 1).

### Retinoid-induced differentiation of EC-derived embryoid bodies is the most efficient method of generation of striatopallidal-like MSNs

**Figure 5:**
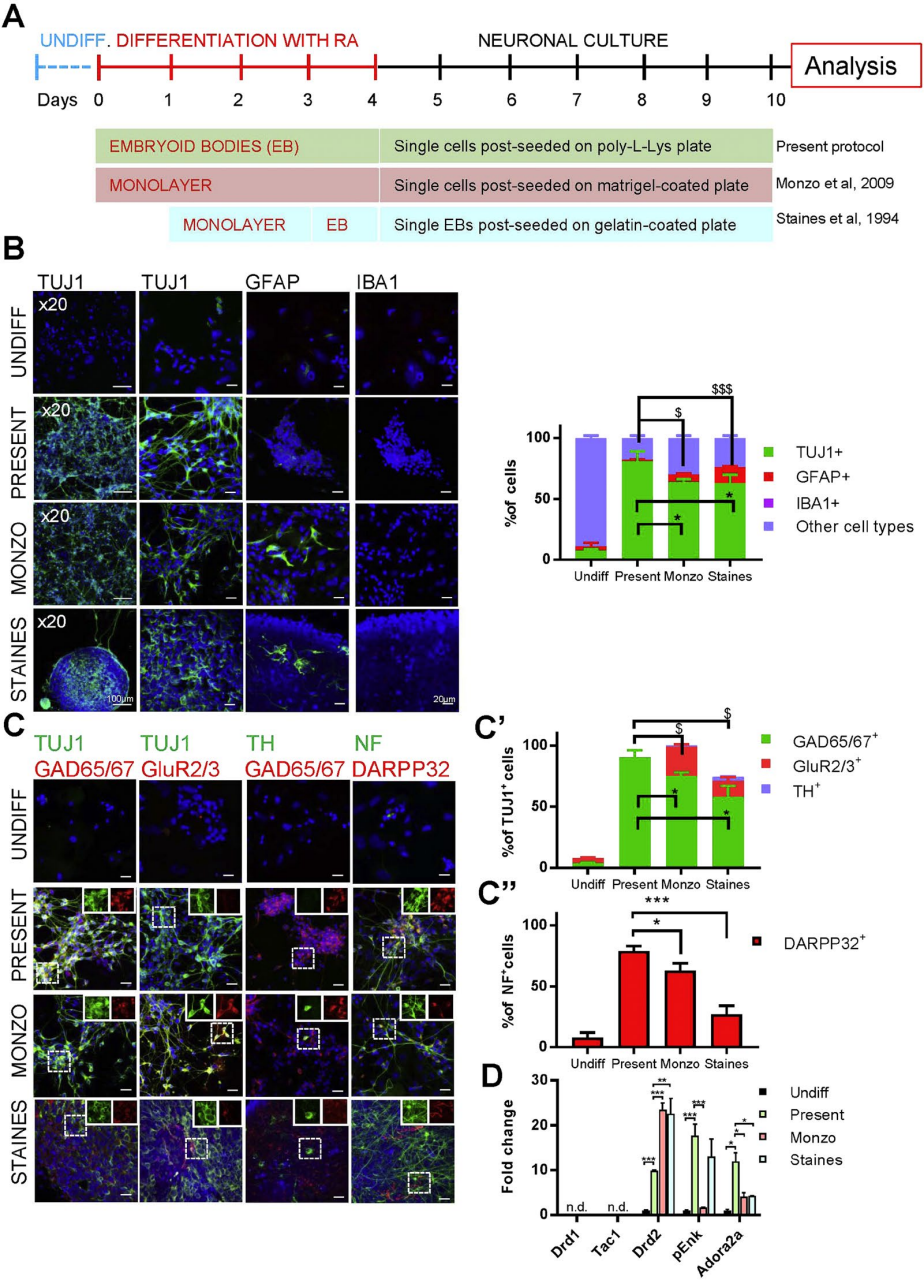
Differentiation of EC cells using distinct protocols. (**A**) Experimental design for neuronal differentiation of EC cells using embryoid bodies (EB), monolayers, and a mixture of EB and monolayers, according to published protocols (Staines *et al*., Monzo et al., see Main text for refs). (**B**) Generation of neurons, astrocytes and microglial cells was assessed using immunocytochemistry for three distinct protocols (Staines, Monzo, present protocol; see examples on left panels and quantifications on the right panel). *p < 0.05 as compared to number of TUJ1^+^ cells generated by  present protocol. ^$^p < 0.05, ^$$$^p < 0.001 as compared to number of GFAP^+^ cells obtained in present protocol. (**C**) Immunofluorescence analysis of neuronal populations: inhibitory (GAD65/67^+^), excitatory (GluR2/3^+^), dopaminergic (TH^+^) and MSN (DARPP32^+^) neurons. (**C’**) Quantification of different types of TUJ1^+^ neurons and (**C”**) NF^+^ MSNs obtained with distinct protocols. *p < 0.05 as compared to number of GAD65/67^+^ neurons obtained by present protocol. ^$^p < 0.05 as compared to number of GluR2/3^+^ neurons generated by present protocol. (**D**) mRNA expression level of striatonigral and striatopallidal markers quantified by RT-qPCR for each protocol. Three independent samples were used for each experimental condition. The expression of each marker is plotted as fold change with respect to undifferentiated cells. ***p < 0.001, *p < 0.05. n.d., not detected. Throughout the figure, graph bars represent means ± s.e.m. Scale bars: 100 µm (x20 panels), 20 µm (others).

In order to compare the efficiency of neuronal differentiation using previously published protocols and our present method, we differentiated P19 cells using the monolayer protocol reported by Monzo *et al*.^36^ and the sequential monolayer/embryoid body protocol reported by Staines *et al*.^38^ (Fig. 5A), and analyzed the cell types generated. The neuronal population identified by TUJ1 staining represented 60 or 63% of all cells for the previously published protocols, which was significantly less than 88% obtained in the present protocol (Fig. 5B). The lesser neuronal population observed through Monzo’s or Staines’ protocols was associated with a higher number of astrocytes, as determined by GFAP expression in 6 and 13% of all cells, respectively (Fig. 5B), compared to 1.5% in our protocol. Neuronal subtypes generated by protocols based on monolayer cultures were more heterogeneous than in our experimental conditions, as 75% GABAergic neurons expressing GAD65/67 were seen alongside with 24% of glutamatergic neurons expressing GluR2/3 and 1% of TH^+^ cells (1%) in the protocol described by Monzo *et al*., whereas the protocol used by Staines *et al*. yielded 63% GABAergic, 13% glutamatergic and 9% TH^+^ neurons (Fig. 5C,C’). Moreover, both protocols were less efficient in generation of MSN-like neurons, as only 63% and 35% neurons were positive for DARPP32 for Monzo’s and Staines’s procedures, as compared to 79% in the protocol employed in this study (Fig. 5C’’). Accordingly, our protocol was the most consistent in generation of striatopallidal-like MSNs as differentiated cells expressed various markers specific to those neurons including Drd2, Adora2a and pEnk, whereas differentiation using Monzo’s or Staines’s protocols efficiently induced expression of Drd2, but not other markers of striatopallidal neurons (Fig. 5D). None of the protocols generated Drd1^+^ neurons, as indicated by absence of Drd1- and Tac1-expressing cells.

## Discussion

We report here that activation of distinct retinoic acid receptors during formation of embryoid bodies from P19 EC cells induces their differentiation to striatal-like GABAergic medium spiny neurons, or inhibitory dopaminergic neurons. Our data confirm original reports on the possibility of generating GABAergic neurons from EC cells^38,56^ and extend such observations at several levels. First, by applying retinoid treatment during embryoid body formation^35^, we show that the rate of neurogenesis (88%) and homogeneity of obtained GABAergic neurons (90% of neurons) are much higher than previously reported (30–70% for neurogenesis and 60% for GABAergic neuron enrichment)^38^. Accordingly, we observed only rare astrocytes and no microglial cells, in contrast to previous reports, as confirmed by a comparative analysis of cells differentiated through the protocols reported by Staines *et al*. and Monzo *et al*.^36,37,39^. The latter two protocols yielded more heterogeneous populations of neurons: in addition to GABAergic neurons, which remained the major type, we observed a substantial percentage (13–24%) of glutamatergic cells. Such differences may result from protocol variations, which included different concentrations of reagents used or duration of embryoid body formation and retinoid treatment, different types of coatings used for plating EC cells, or duration of cultures which spanned from 5–36 days. The possibility of producing MSNs from EC cells was not investigated in the studies of Staines or Monzo and colleagues. Here we show that GABAergic neurons generated from EC embryoid bodies display several characteristics of striatopallidal MSNs, including expression of DARPP32, Adora2a, pEnk and functional Drd2 receptor, whereas they were devoid of Drd1 and Tac1, which are found in striatonigral MSNs, an alternative population of striatal projection neurons. The protocols described by Monzo or Staines and colleagues, employing monolayers, did not provide any consistent signature of striatopallidal MSNs despite strong induction of Drd2 expression. Remarkably, the involvement of ATRA in formation of MSNs was also reported *in vivo* ^16,19,20^. However, whereas *in vivo* studies documented the overall importance of ATRA for production of striatal GABAergic neurons^16^, or the role of RARβ in differentiation of Drd1^+^ striatonigral projection neurons^19,20^, our present data point to the possibility of a retinoid-mediated differentiation of Drd2^+^ striatopallidal projection neurons. Discriminating the involvement of specific RAR subtypes in control of distinct populations of MSNs, as revealed by the present study in EC cells, may encourage further dedicated analyses of RAR functions in the brain. Thus, whereas RARα and RARβ are the major RARs present in LGE, RARγ, which is absent from developing striatum^19,20^ is the major receptor present in undifferentiated EC cells, which contain only low levels of RARα and RARβ (see ref.^34,57^; and Supplemental Fig. 3A).

In order to dissect the contribution of individual RARs to generation of Drd2^+^ MSNs from EC cells, we induced EC differentiation using single and combined treatments with RARα, RARβ or RARγ selective agonists at concentrations optimizing their isotype-selectivity. Several lines of evidence indicate a functionally predominant role of RARγ in such regulation. Similarly, to ATRA, about 90% of neurons generated by RARγ agonist treatment were GABAergic and displayed a molecular signature specific of striatopallidal Drd2^+^ neurons, suggesting that either ATRA or RARγ agonist can be used to generate with high efficiency this neuronal population. In addition, similar, homogeneous populations of striatopallidal-like MSNs were obtained for each compound treatment which included the RARγ agonist (CD666), i.e. CD666 + BMS753 and CD666 + BMS641. Such findings are in line with previous observations of a major role of RARγ in neuronal differentiation of mouse ES cells^57,58^. Interestingly, previous studies reported the potential of RARα agonists in neuronal differentiation of EC cells^34,57^, but did not investigate functional difference between RARα and/or RARγ in generating different neuronal subtypes. Here we show that RARα activation leads to generation of functional dopaminergic neurons. Individual or combined treatments with RARα (BMS753) and RARβ (BMS641) agonists were much less efficient than the RARγ agonist (CD666) or ATRA to generate Drd2^+^ MSNs. However, only RARα and RARβ treatments induced GABAergic neurons expressing TH (the latter never detected after ATRA or CD666 treatment). Such neurons represented about 13% of all cells and 20% of all GABAergic neurons. Expression of dopamine transporter (DAT) indicated that these cells may correspond to a discrete population of dopaminergic neurons which are inhibitory and which in substantia nigra represent about 10% of all TH^+^ neurons^44^. The dopaminergic phenotype of these neurons was also supported by absence of expression of noradrenaline transporter (NET), a marker of noradrenergic neurons which also express TH and production of dopamine by BMS753-generated neurons. Importantly, the efficiency of BMS753 in generation of dopaminergic neurons cannot reflect weak selectivity of RARa agonist and activation of other RAR isotypes, as single of combined treatments with ligands selective for other RARs were not as consistent in generating dopaminergic phenotype.

**Figure 6:**
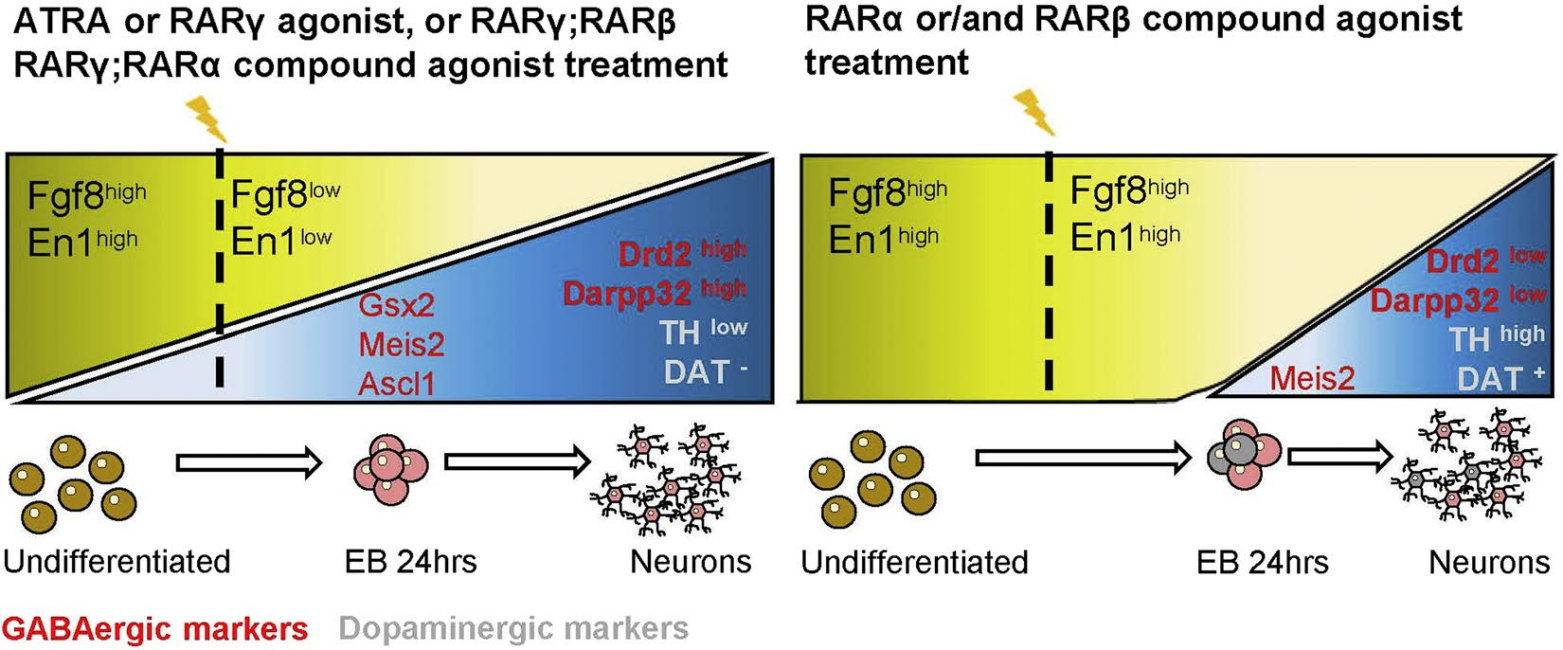
Schematic model of differentiation steps in P19 EC cells. Left-side scheme, expression of striatal determinants (Ascl1, Gsx2) coincide with suppression of midbrain determinants (Fgf8, En1) at 24 h after ATRA or combined RARγ;RARα or RARγ;RARβ agonist treatments, leading to generation of striatopallidal-like MSNs (Drd2^+^, DARPP32^+^). Those neurons do not express TH or DAT. Right-side scheme, EC cells treated with RARα or RARα;RARβ agonists increase or retain high expression levels of midbrain determinants (Fgf8 and En1) with concomitant suppression of key striatal determinants (Ascl1, Gsx2), giving rise to a mixed population of striatopallidal-like MSNs (Drd2^low^, DARPP32^low^) and inhibitory (GAD65/67^+^) dopaminergic neurons (TH^+^ and DAT^+^). In red are indicated GABAergic/striatal markers and dopaminergic markers in yellow.

Altogether, our data suggest that ATRA and specific retinoids activate in EC embryoid bodies a default developmental program of MSN differentiation, which is mostly RARγ-dependent, whereas selective activation of RARα and/or RARβ leads to less efficient MSN formation at the expense of production of DA neurons (Fig. 6). We showed that such programs are activated at the early phase of differentiation (24 h after treatment of EC embryoid bodies), as ATRA and CD666 strongly induced expression of determinants of striatal GABAergic neurons (Ascl1 and Gsx2), whereas RARα or RARβ agonists displayed much weaker induction of the same genes or even their downregulation in the case of compound BMS753 + BMS641 treatment. Instead, RARα and RARβ agonist treatments maintained or enhanced expression of Fgf8 and En1, early determinants of midbrain dopaminergic neurons, which were otherwise strongly downregulated by both ATRA and CD666 treatments. Also striking was the very limited potential of the RARβ agonist to induce EC embryoid body differentiation. This observation was not surprising as a similar, weak differentiation potential of a RARβ agonist was previously observed during differentiation of P19 or F9 cells using different protocols^34,57^, suggesting the possibility of unrelated RARβ functions, e.g. in control of cellular homeostasis as suggested by our recent genomic analyses^59^. Surprising also, was the inefficiency of combined treatment with RARα and RARβ agonists in generating striatal-like MSNs, despite the fact that these receptors are the only RARs being expressed in the developing striatum. We cannot exclude that high levels of RARγ expressed in EC cells may hamper such activities by interacting with a subset of common retinoic acid regulatory elements. In absence of an activating ligand, RARγ would be expected to recruit corepressors and impede transcription of relevant target genes. Such repressive activity of unliganded RARγ on expression of Hoxa1 and RARβ2 transcripts was previously observed in F9 cells, and for RARβ2 also in EC cells^57^. Thus, in order to use EC cells as a model to reveal functions and molecular events controlled by RARα and RARβ during striatal development, one may need to combine pharmacological activation of both receptors with genetic inactivation of RARγ, which should allow reconstitution in EC cells of the pattern of RAR-isotype signaling occuring in LGE. To apply this strategy to investigate retinoid control of neurogenesis in other brain regions, it will be necessary to determine endogenous, cell-type specific repertoires of RAR isotype expression in distinct regions of the developing and adult brain.

The present data encourage detailed studies of the role of specific RAR isotypes in differentiation of ES/iPS cells for regenerative medicine. Transcriptomic comparisons of ATRA-mediated EC and ES cell differentiation revealed important similarities^34,35^. ATRA has been shown to induce transcriptional programs which are common to distinct cell lines and are required for the early phases of cell differentiation including the arrest of cell pluripotency and induction of differentiation^34^. Whereas different RAR isotypes are competent to transduce this early signal, the induction of subprograms necessary for differentiation of specific cell types are dependent on RAR isotypes: RARα (neurons in monolayer-differentiated P19^34,57^ cells or dopaminergic neurons in present study) and RARγ isotypes (endodermal differentiation in F9 cells^34,57^ or MSNs in present study). Thus, low cell-type specificity of ATRA observed during differentiation of ES cells^34,35,60,61^, may reflect simultaneous activation of different RARs, each inducing a different cell type(s). In addition to future phenotypic analyses, identification of common and cell-specific programs induced by during ES/iPS cell differentiation by RAR selective ligands, will be important as it should determine key transcriptional factors or signaling molecules necessary for induction of subnetworks critical for lineage identity. Combinatorial modulation of such critical factors together with RAR-isotype selective retinoids was recently demonstrated as an efficient tool for transdifferentiation of F9 EC to neuronal cells^34^.

In conclusion, there are several potential utilities of the present data. Our study validates an easily accessible protocol of EC cell differentiation as the most efficient method for *in vitro* generation of a highly enriched and homogeneous population of striatopallidal-like, Drd2^+^ MSNs, reaching 90% of all generated neurons. In comparison, the efficiency of generating MSNs using other published protocols varied at best between 40 and 50%^32^. Our EC cell differentiation procedure may be useful for studies of the development and functions of those neurons, as well as functions of the Drd2 dopamine receptor. Our findings are also of interest as this population of neurons is among the most vulnerable to neurodegeneration, as observed in Huntington’s disease, and its dysfunction is associated with physiopathology of Parkinson’s disease, schizophrenia, or depression^21^. RARα/β induction of EC embryoid bodies is also the first model for generation and studies of dopamine-producing GABAergic neurons, whose functions and origin remain unknown despite their identification *in vivo*. The present study provides a functional perspective for interpreting genomic studies of EC cells, and points to their potential utility as a model for studying human and mouse neural stem cells and elaborating stem cell-based strategies in treatment of neurodegenerative diseases.

## Methods

### Cell culture and differentiation

P19 EC cells were propagated undifferentiated and grown in exponential phase in T-75 culture flasks (BD Falcon) using low glucose [1 g/l] Dulbecco’s Modified Eagle Medium (DMEM) containing 5% fetal calf serum (FCS) (Sigma, ref. F-7524) and 5% delipidated fetal calf serum (dFCS) (Jacques Boy), to prevent spontaneous differentiation (Undifferentiation Medium [UM]). Medium was supplemented with 2 mM glutamine and 10 µl/ml gentamycin. After reaching 70–80% confluency, cells were used for induction of differentiation (see below), or were used for further passages. To this end, EC cells were first grown in suspension in P10 Petri dishes (Greiner) for 24 h to separate undifferentiated from differentiating adherent cells, and undifferentiated cells present in suspension were mechanically dissociated and seeded at the density of 400 000 cells per T-75 flask in 20 ml UM. These cells were used as a negative control for differentiated cells. They are referred as undifferentiated in the Figures and in the text.

To induce differentiation, cells were washed twice in phosphate-buffered saline (PBS) and detached with 1 ml of 0.001% trypsin for 1 min. Immediately, 9 ml of UM was added to obtain a cell suspension, followed by 5 min of 1000 rpm centrifugation at room temperature (RT). The supernatant was discarded and the cell pellet was resuspended in α-MEM 1900 (Gibco ref. 22571–038) supplemented with 10% FCS (Differentiation Medium [DM]). 3 $\times$ 10^6^ cells were seeded in P10 Petri dishes in 10 ml of DM, and ATRA, RAR agonist, or a mixture of agonists was added immediately to obtain the following concentrations: 5 µM (ATRA), 100 nM (BMS753), 300 nM (BMS641), 100 nM (CD666). Cell cultures were placed in an incubation chamber at 37 °C (95% O_2,_ 5% CO_2_) for 96 h. First aggregates were visible after 24 h of culture.

After 4 days of ATRA exposure, aggregates (embryoid bodies) were collected by sedimenting in 15 ml conical BD Falcon tubes, washed in PBS, trypsinized in 1 ml of 0,25% Trypsin for 3 min in a 37 °C water bath, and mechanically dissociated using pipetting in 10 ml of DM. The cell suspension was filtered by a 40 µm nylon filter (Sigma, ref. CLS431750-50EA) into 10 ml of DM, and after centrifugation was resuspended in Neuronal Medium (NM) consisting of DMEM (4.5 g/l glucose)-GLUTAMAX-1-Ham-F12 (1:1) medium supplemented with N2 (Gibco, ref. 17502048) and fibronectin (Sigma, ref. F1141). 2.5 $\times$ 10^6^ cells were seeded in 60 mm Petri dishes (BD Falcon) coated with (0.005%) poly-L-lysine (Sigma, ref. P4707). Cells attached immediately, and after 24 h the first dendrites were visible. NM was replaced every third day and at the sixth day cells were collected for analysis (see Fig. 1A).

In order to compare present and previously published experimental conditions we also differentiated P19 cells according to protocols published by Staines *et al*.^38^, and Monzo *et al*.^36^ (see protocol comparison in Fig. 5A). Briefly, using the protocol by Staines *et al*., P19 cells were cultured in T-75 flasks in monolayers for 48 h with 1 µM ATRA and then detached with trypsin, followed by plating in Petri dishes containing freshly made ATRA solution for an additional 24 h. The resulting aggregates were collected by sedimenting and carefully transferred into gelatin-coated plates or glass coverslips in Neuronal Medium (NM) consisting of DMEM (4.5 g/l glucose)-GLUTAMAX-1-Ham-F12 (1:1) medium supplemented with N2 (Gibco, ref. 17502048) and fibronectin (Sigma, ref. F1141), as described above. Medium was changed every third day. Neuronal cells were collected after 6 days. The protocol by Monzo *et al*. was implemented by seeding P19 cells (6 $\times$ 10^4^ cells per cm^2^) in T-75 flasks in MEMα medium with 1 µM ATRA. Cells formed homogeneous monolayers and were incubated for 4 days, with culture medium replacement after the first 48 h. Cells were then trypsinised and 9 $\times$ 10^4^ cells per cm^2^ were seeded in Neurobasal-A medium (NBA) containing 1 $\times$ N2 and 2 mM GLUTAMAX on Matrigel (BD Biosciences)-coated plates. Cell were cultured in NBA medium for 6 days with medium replacement every third day.

### Pharmacological compounds

Synthetic agonists for RARα (BMS753), RARβ (BMS641) and RARγ (CD666) (abbreviated as agoRARα, agoRARβ, agoRARγ in the figures), were synthesized in our laboratories. BMS753 was prepared as described in the original patent^62^. BMS641 was synthesized as reported^63^. CD666 was prepared following the procedure described in the original publication^64^. Haloperidol (Sigma, ref. H-1512) was dissolved in water containing 5% acetic acid and used at 10 µM final concentration. SKF81297 (Tocris, ref. 1447) was prepared in sterile water and used at 1 µM final concentration. Dopamine (Sigma, ref. 8502) was dissolved in darkness in the presence of 500 µM ascorbic acid (Sigma ref., A4403), and used at 1 µM final concentration. Control cells were treated in sterile water.

### Immunocytochemistry (ICC) and cell counts

After embryoid body dissociation, cells were grown for 6 days in 60 mm Petri dishes containing glass coverslips (1 cm diameter) and used for ICC. Cells were fixed in 4% paraformaldehyde (PFA) for 10 min at RT and permeabilized with PBS containing 0,1% Triton X-100 (Sigma, ref. T8787). Non-specific antibody binding was blocked by treatment with 10% FCS for 1 h at RT. Cells were incubated with primary antibodies (see Table 1 below) for 2 h at RT, washed three times with PBS with 0,1% triton X-100 and incubated with secondary antibodies (see Table 1 below). DAPI was used for nuclear counterstained. For imaging, coverslips were mounted with AquaPolyMount (Biovalley, ref. 18606). Cells were imaged using a confocal microscope (SP8UV, Leica) with an 63x objective. Three independent experiments were performed, and at least three coverslips for each condition were examined. Five different random fields per coverslip were analyzed to assess a specific cell population. Cells were counted manually according stereological criteria using the Cell counter plugin in Fiji software.

**Table 1:** Reference and working concentration for primary and secondary antibodies used in fluorescence immunocytochemistry (ICC) and flow cytometry (FACS).

Antibody	Host	Supplier	Cat Nb	ICC	FACS
anti-GAD65/67	Rabbit	Sigma	G5163	1:1000	1:2000
anti-GluR2/3	Rabbit	Chemicon	05–823	1:1000	—
anti-TH	Mouse	Sigma	T2928	1:1000	—
anti-TUJ1βIII	Mouse	Covance	MMS-435P	1:1000	1:200
anti-Iba1	Rabbit	WAKO	019–19741	1:1000	—
anti-GFAP	Mouse	Sigma	G3893	1:1000	—
anti-DARPP32	Rabbit	Millipore	AB1656	1:1000	—
anti-Neurofilament	Mouse	Chemicon	MAB1615	1:1000	—
anti-mouse 488	Donkey	Invitrogen	A21202	1:1000	1:100
anti-rabbit 555	Goat	Invitrogen	A21428	1:1000	1:100

### Flow cytometry (FACS)

Cells were collected by mild trypsinization in FACS buffer (PBS supplemented with 2% FCS), fixed by 1% PFA and permeabilized by 1% saponin, followed by indirect immunostaining with primary antibody detected by a secondary antibody coupled with fluorophore (see Table 1). Only single intracellular staining was performed. Cells were washed in FACS buffer and analysed by FACS Calibur. Cell populations were assessed by FlowJo software.

### Quantitative PCR (RT-qPCR)

Cells were collected at 6 day post-seeding, washed one time in PBS followed by direct cell harvesting with RLT buffer provided with the RNeasy MiniKit (Qiagen, ref. 74104), and RNA was isolated according to the manufacturer’s protocol. Thereafter, cDNA was synthesized with the Transcriptor Kit (Roche, ref. 03531287001). cDNA was diluted 10 times and polymerase chain reaction (PCR) was performed using SybrGreen (Qiagen, ref. 1017340) and 5 µM of each primer (Sigma) (see Table 2). Primers specificity was assessed by melting curve. Quantitative real-time PCR was performed in triplicates and n = 3–4 distinct samples from independent experiments were used for analyses. The expression level for each gene was normalized for expression of housekeeping gene *36B4* (also known as *Rplp0*). Fold change was calculated by ddCt method.

**Table 2:** Sequences of primers used for quantitative PCR.

Gene ID	Forward	Reversed
Drd1	AAGATGCCGAGGATGACAAC	CCCTCTCCAAAGCTGAGATG
Tac1	AGGCTCTTTATGGACATGGC	TCTTTCGTAGTTCTGCATCGC
Drd2	TCGCCATTGTCTGGGTCCTG	TGCCCTTGAGTGGTGTCTTC
Adora2a	CAGAGTTCCATCTTCAGCCTC	CACCCAGCAAATCGCAATG
pEnk	AAAATCTGGGAGACCTGCAA	TCTTCTGGCTCCATGGGATA
Dat	CATGCTGCTCACTCTGGGTA	GTGGTCCAGCAGTGTGAAGA
Net	GGAAAGGAGTGAAGACATCGG	AGGCGGTAGAAGTCAATGTG
Th	AAGATCAAACCTACCAGCCG	TACGGGTCAAACTTCACAGAG
DARPP32	AGGCCTCTCCACATCAGAGA	TCCTCCTCATCATCCTCCTG
Gad67 (Gad1)	TGGACATCTTCAAGTTCTGGC	CTTGGCGTAGAGGTAATCAGC
Gfap	GAAAACCGCATCACCATTCC	CTTAATGACCTCACCATCCCG
CD11b	TCTCAACTTCACGGCTTCAG	TGATCCCATACGGTCACATTG
TUJ1	CGCCTTTGGACACCTATTCAG	TTCTCACACTCTTTCCGCAC
Gsx2	GATTCCACTGCCTCTCCATG	CGGGACAGGTACATATTGGAAG
Meis2	TAGTGCAGCCCATGATTGAC	GGACCACCCTGAGAAACGTA
Meis1	TAACTGACCAGCCCTCTTGG	GTCATCATCGTCACCTGTGC
En1	CTACTCATGGGTTCGGCTAAC	TCTTTAGCTTCCTGGTGCG
Fgf8	CGAAGCTCATTGTGGAGACC	TGTACCAGCCCTCGTACTTG
Ascl1	GACTTGAACTCTATGGCGGG	TTCCAAAGTCCATTCCCAGG
36B4	ACCCTGAAGTGCTCGACATC	AGGAAGGCCTTGACCTTTTC

### High-pressure liquid chromatography (HPLC)

For analyses of dopamine production, dissociated embryoid bodies (7 $\times$ 10^6^ cells) were seeded into poly-L-lysine-coated cell culture dishes (10 mm) in NM. At the 6^th^ day, adherent neurons were trypsinised (0.001% trypsin) and triturated in aMEM medium with 10% FCS. Cells were pelleted (5 min, 1000 rpm) and washed once in PBS. Then cells were transferred into dark eppendrof tubes and spun down to obtain a cell pellet that was immediately frozen at −80 °C. The dopamine content was measured using high performance liquid chromatography with electrochemical detection (HPLC-EC). Briefly, cell pellets were homogenized in ice-cold 0.1 M HClO_4_ and were centrifuged at 10 000 g for 10 min at 4 °C. The supernatant (3–5 µL) was injected into the HPLC system. The chromatography system consisted of an LC-4C amperometric detector with a cross-flow detector cell (BAS, IN, USA), a Ultimate 3000 (Thermo Scientific, USA) pump and a Hypersil Gold analytical column (3 µm, 100 × 3 mm, Thermo Scientific, USA). The mobile phase consisted of 0.1 M KH_2_PO_4_, 0.5 mM Na_2_EDTA, 80 mg/L sodium 1-octanesulfonate, and 4% methanol, adjusted to pH 3.7 with 85% H_3_PO_4_. The flow rate was 1 mL/min. The potential of a 3-mm glassy carbon electrode was set at 0.7 V with sensitivity of 5 nA/V. The temperature of the column was maintained at 30 °C. The Chromax 2007 program (Pol-Lab, Warszawa, Poland) was used for data collection and analysis.

### Statistical analysis

Statistical differences were evaluated using paired Student’s t-test. Differences were considered to be significant when P < 0.05. Calculation and graphs were prepared in GraphPad Prism 7.0. In multiple comparison procedure (one-way ANOVA), the Holm-Sidak method was used for post-hoc analysis.

## Electronic supplementary material

Supplemental information
