# Supplementary material for: Distinct retinoic acid receptor (RAR) isotypes control differentiation of embryonal carcinoma cells to dopaminergic or striatopallidal medium spiny neurons
Source: Sci Rep. 2017 Oct 20;7:13671. doi: 10.1038/s41598-017-13826-x (PMC5651880; doi:10.1038/s41598-017-13826-x)
Supplement: Supplementary file 1 — Supplemental information [file 41598_2017_13826_MOESM1_ESM.pdf]

Distinct retinoic acid receptor (RAR) isotypes control differentiation of embryonal carcinoma cells to dopaminergic or striatopallidal medium spiny neurons

Anna Podleśny-Drabiniok[1,2,3,4,5], Joanna Sobska[1,2,3,4,6], Angel R. de Lera[7], Krystyna Gołembiowska[8], Katarzyna Kamińska[8], Pascal Dollé[1,2,3,4], Małgorzata Cebrat[5], Wojciech Krężel[1,2,3,4*]

[1] Institut de Génétique et de Biologie Moléculaire et Cellulaire, Illkirch, France

[2] Institut de la Santé et de la Recherche Médicale, U964, Illkirch, France

[3] Centre National de la Recherche Scientifique, UMR 7104, Illkirch, France

[4] Université de Strasbourg, Illkirch, France

[5] Laboratory of Molecular and Cellular Immunology, Department of Tumor Immunology, L. Hirszfeld Institute of Immunology and Experimental Therapy, Polish Academy of Sciences, Wroclaw, Poland

[6] Advanced Materials Engineering and Modelling Group, Faculty of Chemistry, Wroclaw University of Science and Technology, Wyb. Wyspianskiego 27, 50-370 Wroclaw, Poland

[7] Departamento de Química Orgánica, Facultade de Química, CINBIO and IIS Galicia Sur. Universidade de Vigo, Vigo, Spain

[8] Department of Pharmacology, Institute of Pharmacology, Polish Academy of Science, Kraków, Poland

SUPPLEMENTAL INFORMATION

SUPPLEMENTAL TABLE 1

	ATRA			agoRARα			agoRARβ			agoRARγ		
	**FC**	*S.E.M.*	p value	**FC**	*S.E.M.*	p value	**FC**	*S.E.M.*	p value	**FC**	*S.E.M.*	p value
**Gsx2**	**82.85**	*17.35*	0.002	**4.97**	*1.29*	0.226	**2.90**	*0.75*	0.533	**57.98**	*5.05*	0.000
**Meis1**	**282.09**	*22.48*	0.000	**21.86**	*4.05*	0.006	**4.38**	*0.81*	0.012	**367.09**	*27.33*	0.000
**Meis2**	**119.22**	*9.25*	0.000	**63.01**	*13.22*	0.002	**16.28**	*3.10*	0.002	**149.17**	*29.59*	0.002
**Ascl1**	**61.46**	*2.34*	0.000	**4.49**	*1.64*	0.079	**2.15**	*0.36*	0.080	**35.18**	*0.24*	0.000
**En1**	**0.12**	*0.01*	0.000	**0.42**	*0.06*	0.002	**0.83**	*0.08*	0.195	**0.11**	*0.01*	0.000
**Fgf8**	**0.03**	*0.01*	0.000	**1.51**	*0.13*	0.022	**2.39**	*0.12*	0.000	**0.06**	*0.02*	0.000

	ago(RARα + RARβ)			ago(RARα + RARγ)			ago(RARβ + RARγ)		
	**FC**	*S.E.M.*	p value	**FC**	*S.E.M.*	p value	**FC**	*S.E.M.*	p value
**Gsx2**	**0.30**	*0.11*	0.374	**37.73**	*3.54*	0.000	**90.00**	*19.00*	0.002
**Meis1**	**26.29**	*9.77*	0.045	**273.74**	*45.30*	0.003	**271.58**	*6.23*	0.000
**Meis2**	**72.38**	*5.02*	0.000	**177.81**	*11.72*	0.000	**180.00**	*20.00*	0.000
**Ascl1**	**1.37**	*0.27*	0.542	**26.69**	*1.54*	0.000	**88.24**	*5.69*	0.000
**En1**	**0.42**	*0.07*	0.003	**0.12**	*0.00*	0.000	**0.10**	*0.01*	0.000
**Fgf8**	**2.08**	*0.28*	0.017	**0.05**	*0.01*	0.000	**0.03**	*0.00*	0.000

SUPPLEMENTAL FIGURE 1

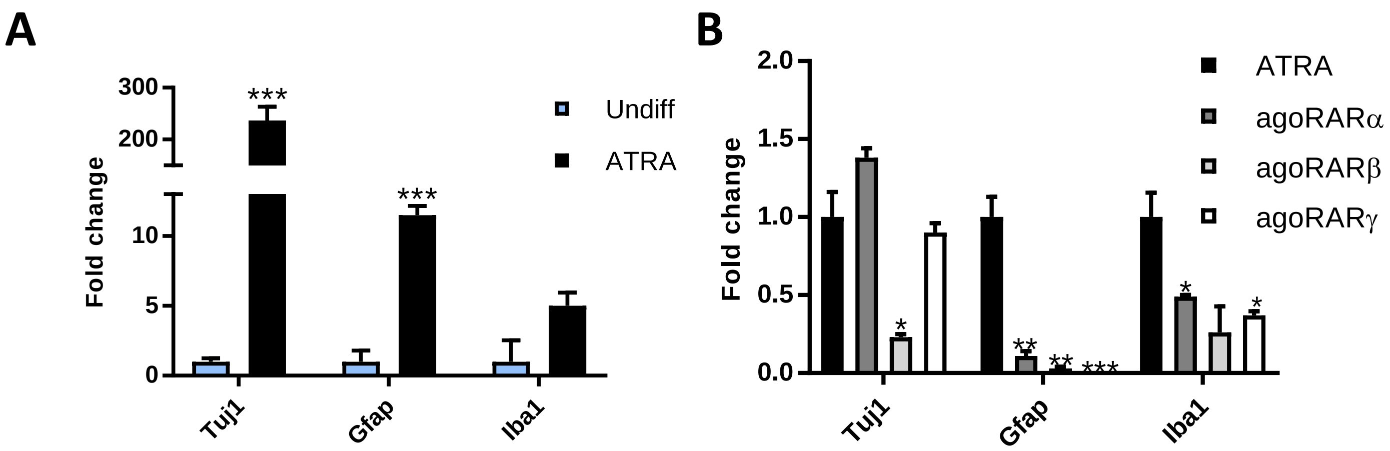

SUPPLEMENTAL FIGURE 2

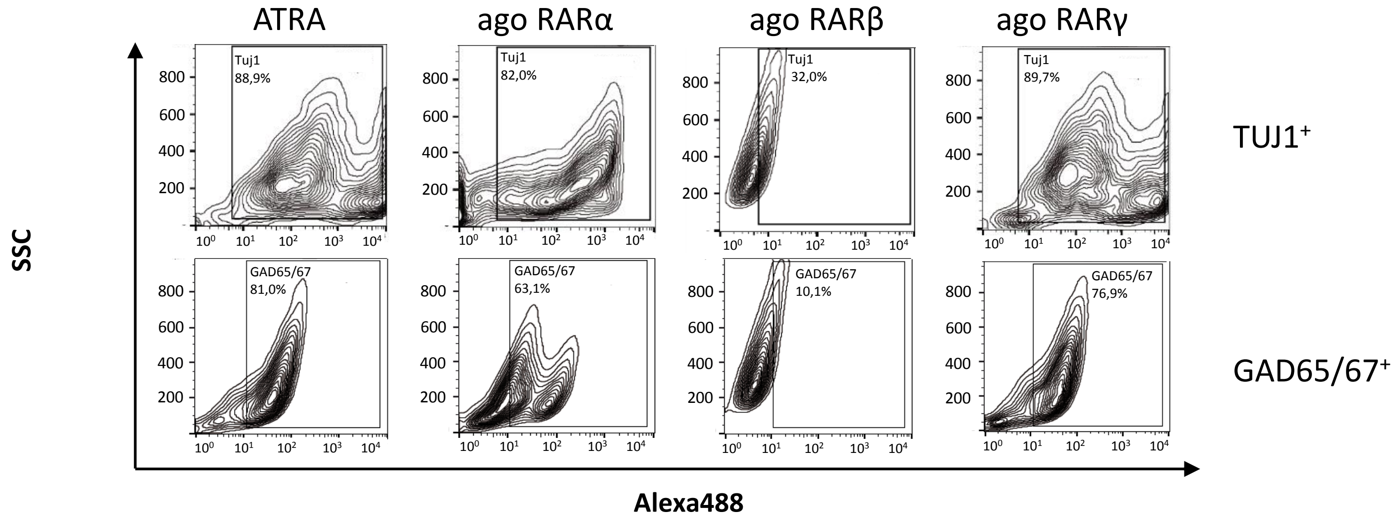

SUPPLEMENTAL FIGURE 3

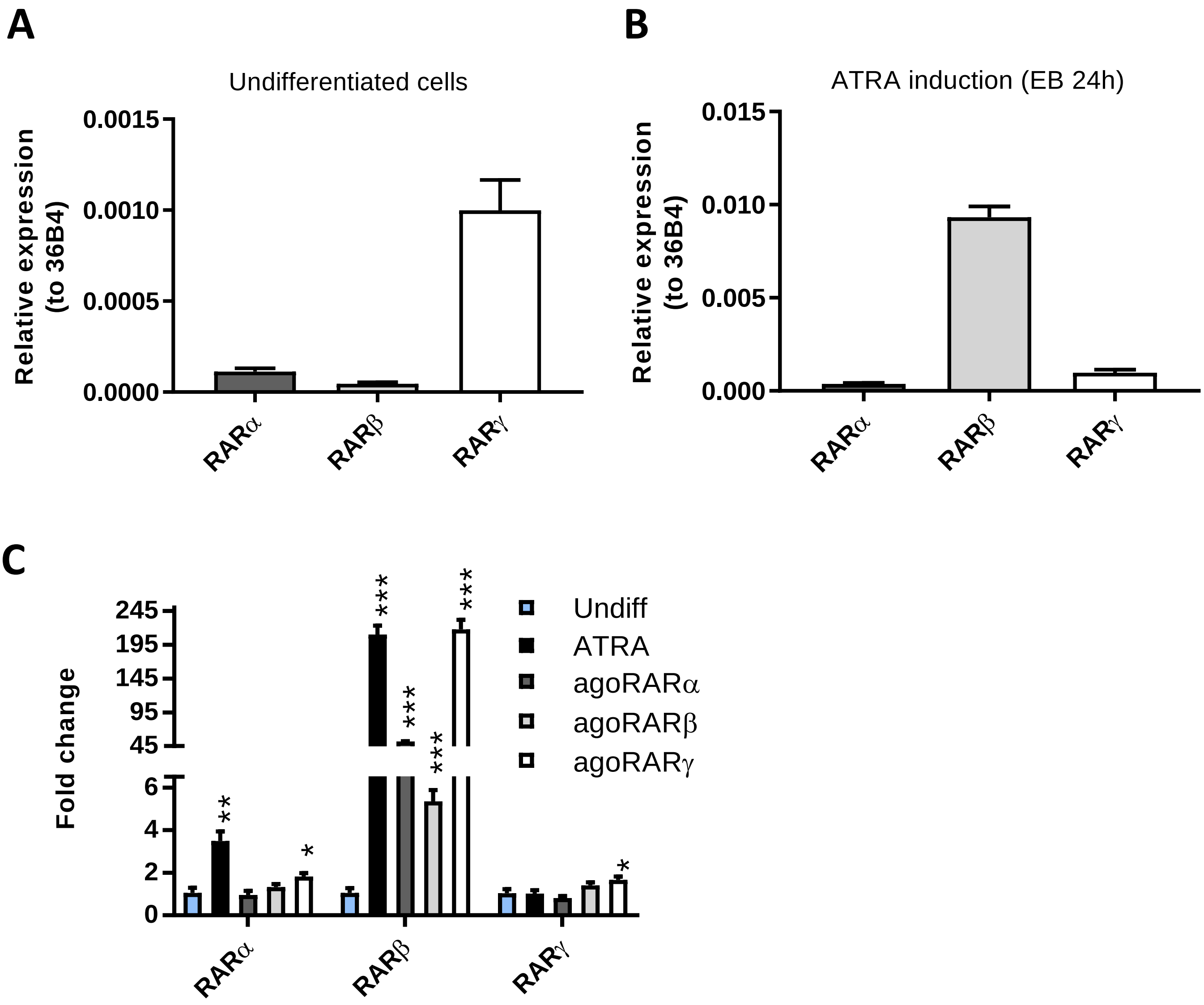

Supplemental figure legends

Supplemental Table 1. (Raw data for Fig. 4). Expression profile of early determinants of GABAergic and dopaminergic neurons in 24h embryoid bodies after distinct retinoic acid receptor (RAR) agonists, Fold changes correspond to the ratio of the mean normalized expression of a particular gene to its mean normalized expression in undifferentiated EC cells (normalized expression meaning $Cp^{gene} – Cp^{housekeeping\ gene}$), n = 3,4 samples per group, p values are obtained through paired Student's t-tests between undifferentiated EC cells and cells treated with RAR agonist(s),

Supplemental Fig.1. Cell types obtained by differentiation of EC cells with all-trans-retinoic acid (ATRA) and selective retinoic acid receptor (RAR) agonists. (A) mRNA expression level for markers of neurons (Tuj1), astrocytes (Gfap) and microglia (Iba1) after ATRA treatment or (B) after treatment with RAR-specific agonists (n=3-4 experiments/treatment). Fold changes (mean ± s.e.m.) were calculated with respect to ATRA treatment. ***$p<0.001$, **$p<0.01$,*$p<0.05$ with respect to ATRA treatment.

Supplemental Fig.2. Neuronal populations obtained after treatment with RAR-selective agonists. Fluorescence-activated cell sorting (FACS) of cells obtained after treatment by distinct RAR agonists. Intracellular labeling for neuronal ($TUJ1^+$) and GABAergic ($GAD65/67^+$) markers followed by detection with a secondary antibody coupled with Alexa488. Contour plots show that neurons were analyzed based on side scatter parameters (SSC) and green fluorescence intensity (Alexa488).

Supplemental Fig.3. Expression of retinoic acid receptors (RAR) in undifferentiated and differentiating EC cells. Relative expression of RARs in (A) undifferentiated cells and (B) EC embryoid bodies at 24h after ATRA treatment (n=3/group). (C) mRNA expression of RARs at 24 h after treatment with ATRA, or specific RAR agonists. Fold changes (mean ± s.e.m.) were calculated with respect to expression in undifferentiated cells (n=3 experiments/treatment). ***$p<0.001$,**$p<0.01$,*$p<0.05$ with respect to ATRA treatment.
